# Evaluation of Probiotic Properties and Safety of *Lactobacillus helveticus* LH10 Derived from Vinegar through Comprehensive Analysis of Genotype and Phenotype

**DOI:** 10.3390/microorganisms12040831

**Published:** 2024-04-19

**Authors:** Yang Du, Jingru Xu, Jinquan Li, Renwei Wu

**Affiliations:** College of Food Science and Technology, Huazhong Agricultural University, Wuhan 430070, China; duy2333@163.com (Y.D.); xu0208food@163.com (J.X.); lijinquan2007@gmail.com (J.L.)

**Keywords:** *Lactobacillus helveticus*, whole-genome sequencing, phenotypic analysis, safety and probiotic properties, cereal vinegar

## Abstract

The probiotic potential of *Lactobacillus helveticus* LH10, derived from vinegar Pei, a brewing mixture, was assessed through genotype and phenotype analyses. The assembled genome was comprised of 1,810,276 bp and predicted a total of 2044 coding sequences (CDSs). Based on the whole genome sequence analysis, two bacteriocin gene clusters were identified, while no pathogenic genes were detected. In in vitro experiments, *L. helveticus* LH10 exhibited excellent tolerance to simulated gastrointestinal fluid, a positive hydrophobic interaction with xylene, and good auto-aggregation properties. Additionally, this strain demonstrated varying degrees of resistance to five antibiotics, strong antagonistic activity against four tested pathogens, and no hemolytic activity. Therefore, *L. helveticus* LH10 holds great promise as a potential probiotic candidate deserving further investigation for its beneficial effects on human health.

## 1. Introduction

Lactic acid bacteria have emerged as the most extensively consumed probiotics, encompassing not only live bacteria but also postbiotics such as bacterial lysates, metabolites, and non-viable cells that offer a wide range of physiological benefits [1]. The strain-specific nature of probiotic effects has been widely acknowledged [2,3]. In 2022, the National Health Commission of the People’s Republic of China (NHCPRC) issued a notice updating “The list of strains approved for use in food for infants and young children”, which included 13 strains from four genera of lactic acid bacteria: *Lactobacillus helveticus* R0052, *Bifidobacterium animalis* subsp. lactis Bb-12, *Lacticaseibacillus rhamnosus* HN001, and *Limosilactobacillus reuteri* DSM 17938, among others. *Lactobacillus helveticus* has been reported to contribute bioactive peptides with antihypertensive, antimicrobial and antioxidant properties [4], immunomodulatory properties [5], etc.

In recent years, Chinese researchers have been actively exploring the potential of traditional fermented foods as a source for probiotics and have successfully isolated several exceptional strains, including *Bifidobacterium animalis* subsp. lactis Probio-M8 [6], *Lactobacillus plantarum* 4–10 [7], *Latilactobacillus sakei* [8], *Pediococcus pentosaceus* JS35 [9], and *Lactobacillus plantarum* 430 [10]. These efforts have not only contributed to the advancement of pure strain utilization in the food industry but have also served to preserve valuable probiotic resources within their natural environment. Vinegar has long been recognized for its diverse physiological effects, primarily attributed to its unique brewing microbiome composition [11]. Zhenjiang aromatic vinegar, one of China’s four types of grain vinegar produced through solid-state fermentation, is particularly noteworthy. Within this vinegar Pei, *Lactobacillus helveticus* has been identified as a key microorganism responsible for producing bioactive compounds with significant health benefits, including antimicrobial, antidiabetic, antioxidative, antiobesity, and antihypertensive effects [12,13].

Currently, the integration of whole-genome sequencing and bioinformatics could comprehensively explore the genetic and biological characteristics of strains with enhanced resolution and sensitivity. This approach provides valuable insights into various aspects such as genetic information, evolutionary relationships, physiological traits, probiotic potential, safety profiles, among others [14]. However, it is crucial to acknowledge that phenotypic analysis remains indispensable for probiotic discovery due to potential errors in the sequencing process and limited concordance between genomic sequences and existing databases [15].

In this study, a combination of phenotypic experiments and whole-genome sequencing was employed to evaluate the safety and probiotic properties of *L. helveticus* LH10 derived from vinegar Pei, a brewing mixture with alcohol mash, wheat bran, and chaff, as a potential probiotic.

## 2. Materials and Methods

### 2.1. Bacterial Strain and Growth Conditions

*Lactobacillus helveticus* LH10 was isolated from the vinegar Pei provided by the Zhenjiang Vinegar Co. (Zhenjiang, China). Zhenjiang balsamic vinegar follows traditional continuous solid-state brewing with a century-long brewing history in China. The strain was stored at −80 °C until use.

### 2.2. Extraction of Genome DNA

*Lactobacillus helveticus* LH10 was cultured at 37 °C in MRS medium (de Man Rogosa Sharpe) comprised of 10 g/L peptone, 8 g/L beef extract powder, 4 g/L yeast extract powder, 20 g/L glucose, 2 g/L K_2_HPO_4_, 2 g/L C_6_H_14_N_2_O_7_, 5 g/L sodium acetate, 0.2 g/L MgSO_4_, 0.04 g/L MnSO₄, 17 g/L agar, and 1 g/L tween-80 at 37 °C. Genomic DNA was extracted by the SDS method [16]. After extraction, the total DNA was quantified by a Qubit^®^ 2.0 Fluorometer (Thermo Scientific, Boston, MA, USA). The integrity of the DNA was determined by electrophoresis on 1% agarose gels.

### 2.3. Genome Sequencing, Assembly, and Annotation

The whole genome of *L. helveticus* LH10 was sequenced using the Nanopore PromethION platform and Illumina NovaSeq PE150 at the Beijing Novogene Co., Ltd. (Beijing, China). Libraries for Nanopore sequencing were constructed with an insert size of 10 kb. First, large fragments of DNA were recovered by a Blue Pippin automatic nucleic acid fragment recovery system, and then repaired. Barcodes were added by the PCR-free method of an EXP-NBD104 kit (Oxford, UK) of the Oxford Nanopore Technologies Company. Afterwards, the sizes of the fragments were detected by an AATI automatic capillary electrophoresis instrument (Agilent, Co., Beijing, China), and the samples were isomolar-mixed. Finally, an SQK-LSK109 connection kit (Oxford, UK) was used to link the adapter, and the 10K library was constructed. For Illumina Library construction, the DNA sample was fragmented to a size of 350 bp by sonication, then DNA fragments were end-polished, A-tailed, and ligated with the full-length adaptor for Illumina sequencing with further PCR amplification. At last, PCR products were purified (AMPure XP system (Beckman Co., Shanghai, China)) and libraries were analyzed for size distribution by an Agilent 2100 Bioanalyzer (Agilent, Co., Beijing, China) and quantified using real-time PCR. Unicycler (version: 0.4.8) was utilized to combine PE150 data with Nanopore data to assemble, then we compared the reads to the assembled sequence, counted the distribution of the sequencing depth, and distinguished whether the assembled sequence was a chromosomal or plasmid sequence according to sequence length and alignment, and checked whether it was a circular genome.

Coding gene prediction of the sequenced genomes was performed with GeneMarkS (Version 4.17) [17]. Non-coding RNAs (including tRNAs, rRNAs, and sRNAs), genomic island, CRISPR, and prophages were predicted by tRNAscan-SE (Version 1.3.1) [18], rRNAmmer (Version 1.2) [19], the Rfam database [20], IslandPath-DIOMB (Version 0.2) [21], and phiSpy (Version 2.3) [22].

Gene Ontology (GO), the Kyoto Encyclopedia of Genes and Genomes (KEGG), the Cluster of Orthologous Groups of proteins (COG), the Non-Redundant Protein Database (NR), the Transporter Classification Database (TCDB), and the Virulence Factors of Pathogenic Bacteria (VFDB) and Comprehensive Antibiotic Research Database (CARD) were utilized to realize the functional annotation of the whole LH10 genome.

### 2.4. Evolutionary Position

The 16S rRNA sequence of *L. helveticus* LH10 was obtained from the whole genome using RNAmmer. Eight species of *Lactobacillus* were screened from the NCBI database (http://www.ncbi.nlm.nih.gov (accessed on 16 April 2024)) for the construction of the 16S-rRNA-based phylogenetic tree based on the principles of closeness of kinship, different publication year, and strain origin. The phylogenetic tree was constructed by the maximum likelihood method after ClustalW’s multiple comparisons of *L. helveticus* LH10 and related strains using MEGA11.

### 2.5. Average Nucleotide Identity Analysis

Average nucleotide identity (ANI) is a measure of genomic similarity between the coding regions of two genomes at the nucleotide level and a common method used to assess genetic diversity [23]. ANI analyses of different strains were calculated using CJ Bioscience’s OrthoANI Tool (https://www.ezbiocloud.net/tools/ani (accessed on 16 April 2024)), and a heatmap was plotted by OmicShare tools (www.omicshare.com/tools (accessed on 16 April 2024)).

### 2.6. Collinearity Analysis

In this study, *L. helveticus* R0052, an approved probiotic strain isolated from sweet acidophilus milk [24], was selected for collinearity analysis with *L. helveticus* LH10. The genomic comparison software ProgressiveMauve (Version 2.4.0) [25] was used for genomic comparison (default parameter settings) to derive the genetic rearrangements, deletions, and structural differences.

### 2.7. Safety Evaluation of Lactobacillus Helveticus LH10

The antibiotic sensitivity of *L. helveticus* LH10 was determined by the disk diffusion test on MRS agar according to the Clinical and Laboratory Standards Institute guidelines (CLSI) [26]. The disks used gentamicin (10 μg), compound sulfamethoxazole (including trimethoprim, 25 μg), erythromycin (15 μg), penicillin (10 μg), vancomycin (30 μg), tetracycline (30 μg), clindamycin (2 μg), ampicillin (10 μg), ciprofloxacin (10 μg), cefamezin (30 μg), rifampicin (5 μg), and chloramphenicol (30 μg). The plates were incubated at 37 ℃ for 24 h, and then the diameter of the inhibition zone was measured.

Antibiotic resistance genes of *L. helveticus* LH10 were predicted using the Comprehensive Antibiotic Resistance Database (CARD). The amino acid sequences of LH10 were aligned with the Comprehensive Antibiotic Resistance Database (CARD) using Resistance Gene Identifier (RGI) software (v 5.1.0) [27] (RGI built-in blastp, default value ≤ 1 × 10^−30^, similarity > 30%), and the annotated resistance gene information was statistically calculated based on the alignment results of RGI.

According to the virulence factor database (VFDB) annotation of the whole genome of *L. helveticus* LH10, the similarity of the comparison results was set at ≥50% [28].

*L. helveticus* LH10 was inoculated in Columbia agar medium with 7% sterile defibrinated sheep blood and cultured at 37 °C for 24 h. The hemolytic ring was observed to determine the hemolytic activity of the strain [29].

### 2.8. In Vitro Simulated Gastric and Intestinal Digestion

The tolerance of *L. helveticus* LH10 to bile salts and intestinal fluid was investigated as described by Mulaw (2019) [30] and Chen (2017) [31], respectively. Viable bacteria were counted to assess their ability to tolerate gastric fluid after being incubated for 3 h in simulated gastric fluid (35 mg/mL pepsin solution with pH 3). The bacteria were harvested from gastric juice and then incubated at 37 °C for 8 h in simulated intestinal fluid (dissolve 0.1 g trypsin and 1.8 g bile salt in 100 mL distilled water containing 1.1 g NaHCO_3_ and 0.2 g NaCl, and then adjust pH to 8 with NaOH). Samples were collected at intervals of 2 h and viable counts were performed to determine the tolerance of *L. helveticus* LH10 to the intestinal fluids.

### 2.9. Binding Properties

For the hydrophobicity test, *L. helveticus* LH10 was grown in MRS broth and harvested by centrifugation at 5000× *g* for 10 min and then washed twice in PBS (Phosphate Buffer Solution, pH 7.2). Afterward, optical density at 600 nm (OD_600_) of the suspension in PBS was adjusted to 0.8–1.0 (A_0_) by a UV1800 spectrophotometer (Shimadzu Co., Kyoto, Japan). The same volume of bacterial suspension and xylene was thoroughly mixed with a benchtop vortex mixer for 2 min. The aqueous phase was carefully separated, and its OD_600_ was read (A1) [32]. The cell surface hydrophobicity (H%) was calculated as follows,

H% = (1 − A_1_/A_0_) × 100 (1)

For auto-aggregation, the bacterial suspension of *L. helveticus* LH10 was obtained according to the above method, and its OD_600_ was measured (A_0_). The suspension was incubated at 37 °C for 3 h, 6 h, and 9 h, respectively, and we read its OD_600_ (A3-6-9). Auto-aggregation was determined in percentage as follows [33],

Auto-aggregation (%) = (1 − A_(3-6-9)_/A_0_) × 100 (2)

### 2.10. Analysis of Bacteriostatic Properties

Bacteriocin-producing gene clusters were predicted by BAGEL4 (http://bagel4.molgenrug.nl/ (accessed on 16 April 2024)), and the inhibitory effect of *L. helveticus* LH10 on pathogens was verified by the Oxford cup double-layer agar diffusion method [34].

## 3. Results and Discussion

### 3.1. Genome Properties

**Figure 1:**
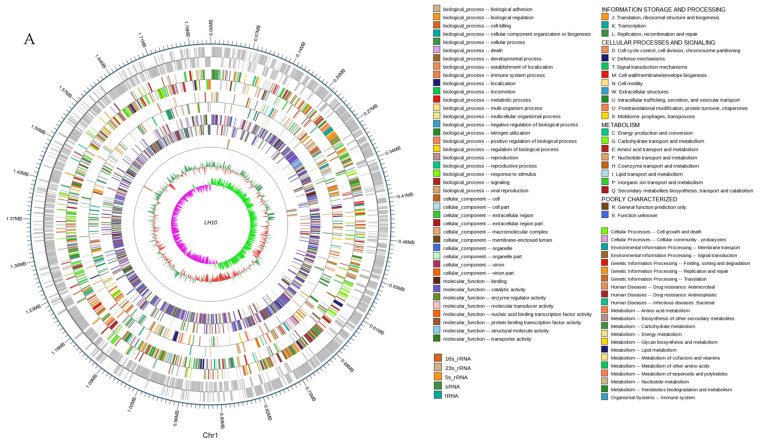

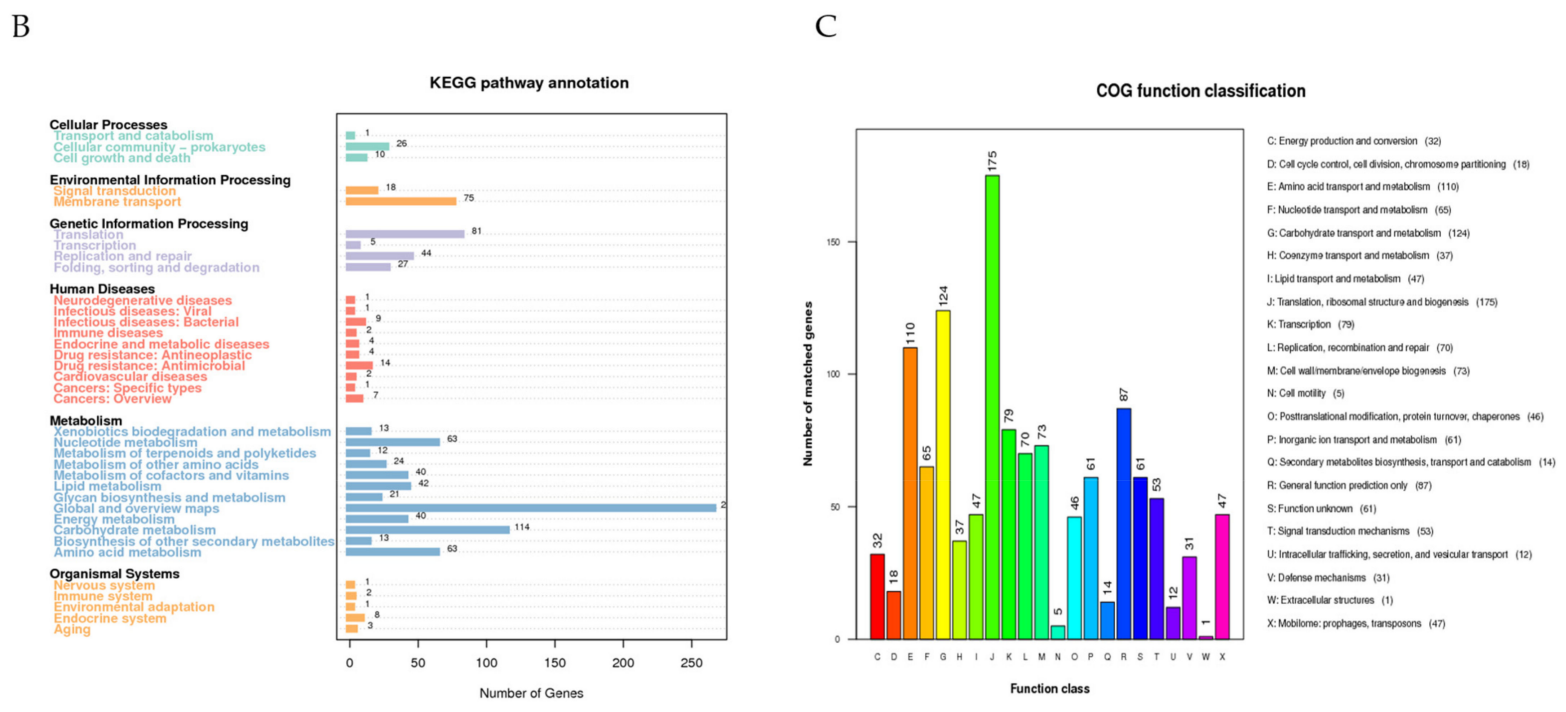

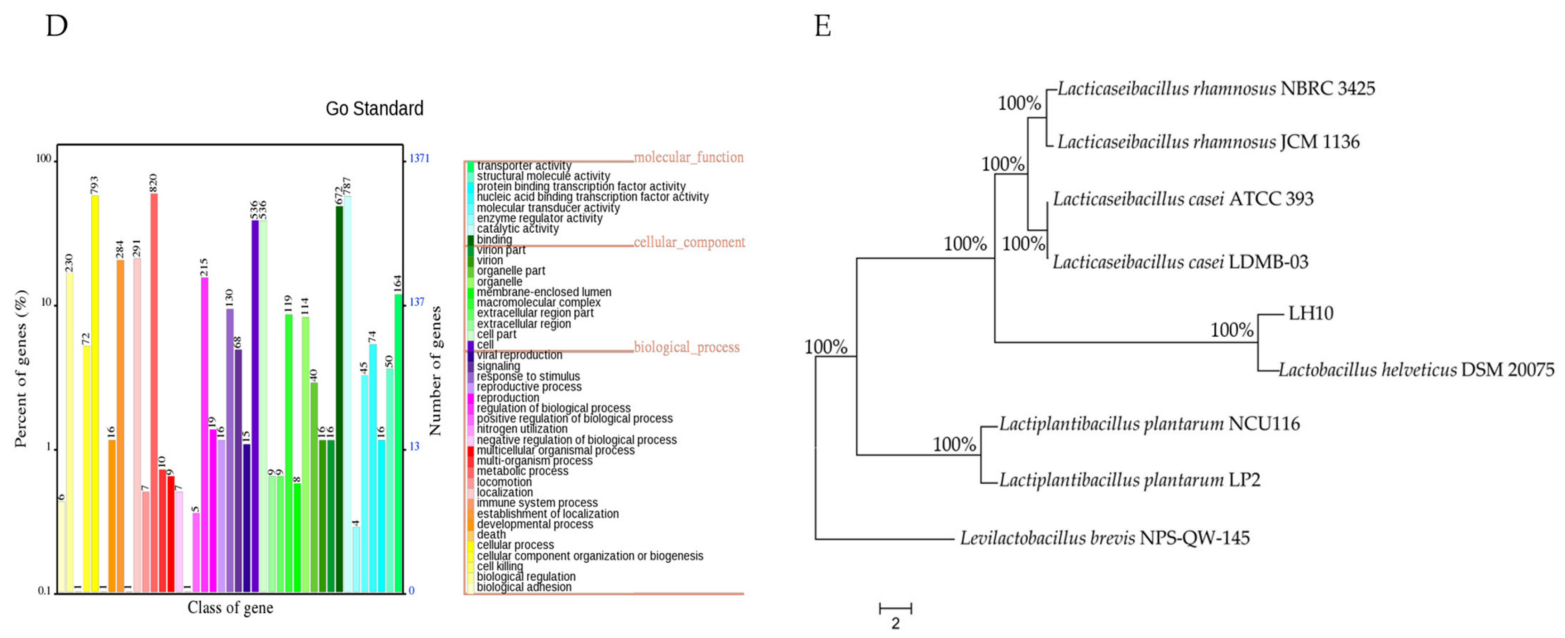
Circular map of the LH10 chromosome and functional annotation of the LH10 genome with different databases. (**A**) From the outside to the inside, the circles represent the coding genes, gene function annotation results (including the annotation information of COG, KEGG, and GO databases), ncRNA, GC content, and the GC skew value, respectively. (**B**) KEGG database classification statistics, (**C**) COG database classification statistics, (**D**) GO database classification statistics, (**E**) Phylogenetic tree of *L. helveticus* LH10 based on 16s rRNA sequences. Approximately 1500 bp were included in the 16S rRNA analysis and 500 bootstrap replications were employed in generating the tree.

The genome sizes of most *Lactobacillus helveticus* strains were mostly above 2 Mb (Table 1), such as *Lactobacillus helveticus* D75 (2.05 Mb), *Lactobacillus helveticus* LH5 (2.16 Mb), and *Lactobacillus helveticus* R0052 (2.13 Mb). However, the genome size of LH10 was only 1.81 Mb, which was significantly shorter than most *Lactobacillus helveticus* strains. In addition, some *Lactobacillus helveticus* strains like *L. helveticus* D75 had 15 rRNAs and *L. helveticus* LH5 had two plasmids, while LH10 only had 12 rRNAs and no plasmids. We also detected four prophages in the LH10 genome. The sizes of the four prophages in the LH10 genome were 11,066 bp, 25,216 bp, 33,357 bp, and 46,488 bp, respectively, and given that the genome sizes of phages are generally above 40 kb [35], it is hypothesized that there is one potential complete prophage genome in LH10. The chromosome circle diagram integrating genomic GC content, GC skew values, ncRNA, and genomic functional annotation is shown in Figure 1A. In Figure 1B, a total of 1836 coding genes were annotated in the KEGG database, accounting for 89.8% of all the coding genes. They were divided into six major categories and 36 subcategories, including cellular processes (2%), environmental information processing (5%), gene information processing (8.5), human diseases (2.4%), metabolism (81.3%), and organismal systems (0.8%). Among them, 1142 and 1172 coding genes were annotated in the COG (Figure 1C) and GO databases (Figure 1D), respectively. The genome sequencing data for *L. helveticus* LH10 were submitted to GenBank (no. CP149445.1).

### 3.2. Phylogenetic Tree

In this study, we used the near-origin species *Levilactobacillus brevis* NPS-QW-145 as an outgroup and constructed the phylogenetic tree by the maximum likelihood (ML) method based on the 16S rRNA sequences. As the result showed, the LH10 strain had the highest sequence identity with *Lactobacillus helveticus* DSM20075, which showed pronounced adaptability to the gut environment and possessed multiple genes encoding bacteriocins [36]. Moreover, they showed the longest evolutionary distance to form a unique branch with other species (Figure 1E).

### 3.3. Average Nucleotide Identity Analysis

**Figure 2:**
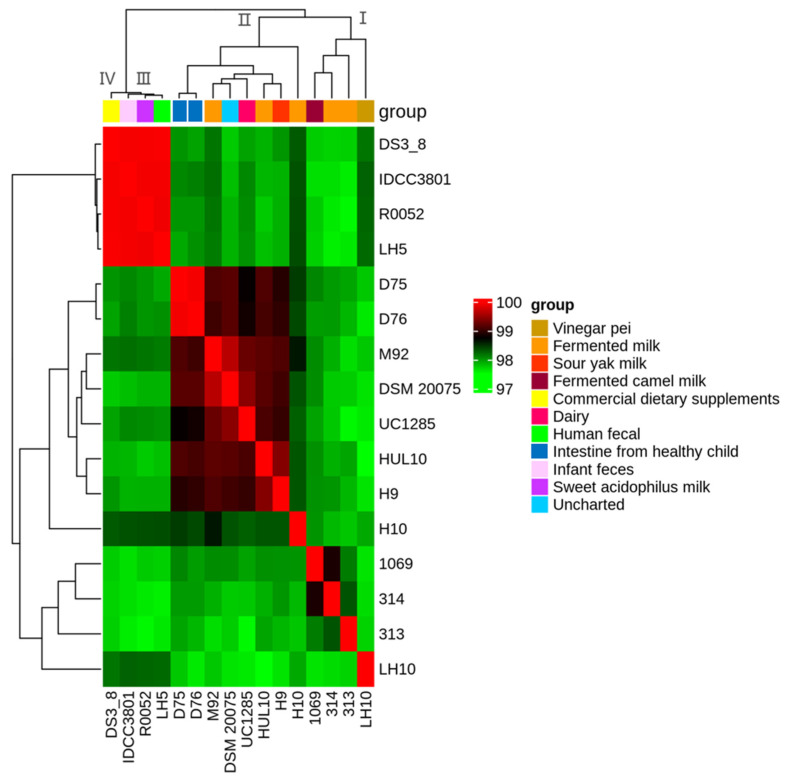
ANI heatmap of 16 *Lactobacillus helveticus* strains. Each cell represents the OrthoANI values between the row and the corresponding genomes of the column.

Most of the currently reported *Lactobacillus helveticus* strains were isolated from dairy products and human intestine, whereas the *Lactobacillus helveticus* LH10 strain was from vinegar Pei. In this study, the genomic homology of *Lactobacillus helveticus* from different sources were further explored (Figure 2). The ANI values among genomes of 16 *Lactobacillus helveticus* were all greater than 97%. According to the distribution of ANI values, 16 *Lactobacillus helveticus* strains could be classified into four taxa: taxon I, taxon I, taxon II, and taxon IV. The heatmap had a noticeable color gradient from the top left to bottom right. Strains from human feces and sweet acidophilus yogurt showed very high ANI values (>99.9%) within taxa III and IV; similarly, eight strains from human and dairy sources remained highly congruent within taxa II. The results did not show a classification based on isolated sources, suggesting that *Lactobacillus helveticus* from different sources may still have high sequence similarity. *L. helveticus* LH10 was classified as taxon Ⅰ and had the highest ANI values with *L. helveticus* IDCC3801 (98.27%) and *L. helveticus* LH5 (98.25%), which were, respectively, from human feces and infant feces. Meanwhile, the ANI values of the other strains were all less than 98%. *L. helveticus* LH10 formed an independent branch, which indicated that the genome of the vinegar-derived *L. helveticus* LH10 had a significant alteration during the evolutionary process.

### 3.4. Collinearity Analysis

**Figure 3:**
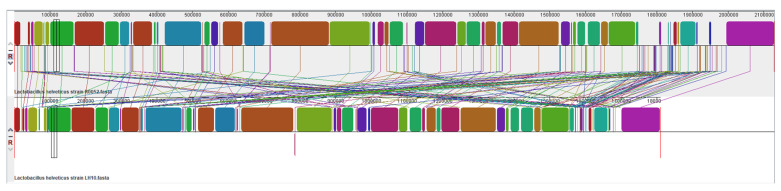
Locally collinear blocks between *L. helveticus* R0052 and *L. helveticus* LH10. Each contiguously colored region is a locally collinear block (LCB), a region without a rearrangement of homologous backbone sequences. LCBs below a genome’s center line are in the reverse complement orientation relative to the reference genome. Lines between genomes trace each orthologous LCB through every genome. Regions outside blocks lack detectable homology among the input genomes.

Collinearity analysis was used to study genomic correlation by detecting the consistency of homologous sequences and their order among genomes of different species or strains. The higher genomic correlation, the higher the consistency of homologous sequences [37]. In this collinearity analysis, the genome of *L. helveticus* R0052, an approved probiotics strain, was used as a reference. *L. helveticus* LH10 and *L. helveticus* R0052 had a total of 73 locally collinear blocks, of which the minimum weight was 46. Compared with the R0052 strain, *L. helveticus* LH10 had one light-weight inverted block (Figure 3), and the genes in the inverted parts were *sufD* (Fe-S cluster assembly protein SufD) and *sufC* (Fe-S cluster assembly ATP-binding protein). In addition, the LH10 genome had a unique segment of 4 kb, mainly for various putative proteins. Meanwhile, *Lactobacillus helveticus* LH10 had significantly fewer empty regions than *Lactobacillus helveticus* R0052, and some homologous blocks of *Lactobacillus helveticus* LH10 underwent simplification. The above results suggested the genome of *L. helveticus* LH10 was more compact.

### 3.5. Antibiotic Susceptibility

Twelve different antibiotics were selected for the susceptibility test of *L. helveticus* LH10. The diameter of the inhibition zone was measured, and the results were expressed as resistant, intermediate, or susceptible according to the CLSI. Of the 12 antibiotics tested (Table 2), *L. helveticus* H10 was sensitive to 6 antibiotics (clindamycin, penicillin, tetracycline, erythromycin, rifampicin, and chloramphenicol), intermediate to 1 antibiotic (ampicillin), and resistant to 5 antibiotics in different degrees (vancomycin, sulfamethoxazole, gentamycin, ciprofloxacin, and cefamezin). This result was consistent with many reports that some lactic acid bacteria are naturally resistant to vancomycin and amino glycosides, which is considered a non-transmissible natural resistance [38,39]. In addition, erythromycin exhibited strong inhibitory activity against LH10, with a bacteriostatic circle diameter of 32 ± 0.3 mm. Further studies are required to determine the minimum inhibitory concentration of the evaluated antibiotics and assess the molecular characterization of the antimicrobial resistance genes to determine the likelihood of being transmitted. Several studies have highlighted that probiotics with specific antibiotic resistances could be useful to be co-administered with an antibiotic therapy because they help maintain the stability of the intestinal flora, preserve the intestinal barrier, and reduce pathogens [40].

### 3.6. Antibiotic Resistance Genotype

Several antibiotic resistance genes were annotated in the LH10 genome by the Comprehensive Antibiotic Research Database (CARD), including macrolide, glycopeptide, tetracycline, peptide, phosphonic acid, aminoglycoside, lincosamide, and rifamycin (Table 3). In the phenotypic experiments, *L. helveticus* LH10 was sensitive to macrolide, tetracycline, and lincosamide. However, some of the predicted resistance genes also exhibited beneficial properties, such as the efflux pump complex gene *mdtG* described as having phosphate resistance, which was also a member of the MFS family of transporter proteins in the KEGG annotation and contributed a positive tolerance to bile salt.

Plasmids are replicable DNA elements located outside the chromosome within the bacterial genome and are the primary mechanism for the spread of antimicrobial resistance [41]. According to gene sequencing results, *L. helveticus* LH10 did not possess plasmids; therefore, its resistance genes could not horizontally transfer.

### 3.7. Hemolytic Activity Test

**Figure 4:**
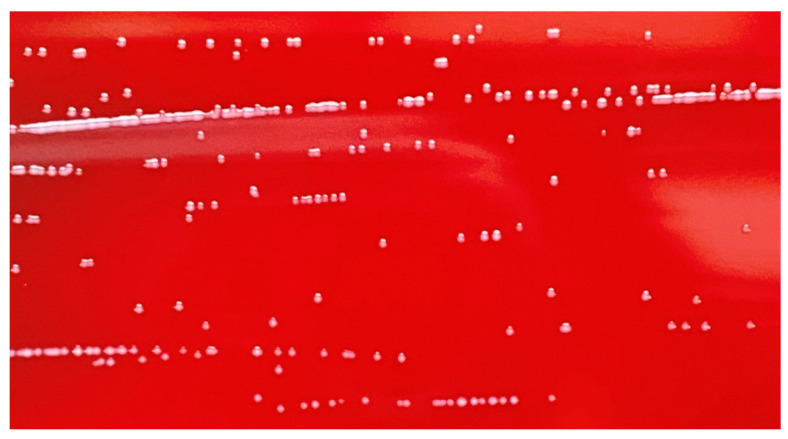
Hemolytic activity analysis of *L. helveticus* LH10 with blood agar plates.

Hemolysis refers to red blood cell rupture and hemoglobin release from the cells. Strains with hemolytic properties can cause sepsis. The hemolytic phenomenon is mainly classified into three categories: the grass-green hemolytic ring (α-hemolysis), colorless transparent hemolytic ring (β-hemolysis), and no hemolytic ring (γ-hemolysis). According to the test (Figure 4), *L. helveticus* LH10 exhibited γ-hemolysis; therefore, it could be considered a safe strain.

### 3.8. Virulence Factors of L. helveticus LH10

According to the annotation of the VFDB, 26 virulence factors were annotated. However, the similarities between these genes and those in the VFDB database were mostly lower than 60%. According to the annotation of the other functional databases, such as the KEGG and COG databases, the prediction of potential virulence genes revealed that most of these genes were annotated as sugar metabolism, nucleotide metabolism, environmental information processing, secondary metabolite biosynthesis, and adhesion. Meanwhile, the annotation of the VFDB may not fit the range of the virulence factors for *Lactobacillus*, such as stress proteins related to environmental stress, which could adapt to a variety of stressful conditions in the human gut and improve survival. In addition, the elongation factor thermo-unstable (*EF-Tu*) plays an important role in protein processing for prokaryotes, thus it also did not belong to the category of virulence factors. Therefore, *L. helveticus* LH10 likely was not capable of producing toxic and harmful metabolites at the genetic level. The *L. helveticus* LH10 genome lacked the genes encoding hemolysin BL(Hbl), non-hemolytic enterotoxin (Nhe), and emetic toxin (cereulide—Ces) [42], which was consistent with the hemolytic activity test in the phenotype experiment. More phenotyping experiments are needed to verify whether LH10 is completely safe for humans.

### 3.9. Binding Properties

Cell surface hydrophobicity is an important feature associated with cell adhesion that provides a competitive advantage for lactic acid bacteria adhesion and colonization in the human gut [43]. The hydrophobicity of *L. helveticus* LH10 was investigated using xylene, and the hydrophobic rate to xylene was 61.38%. In addition, auto-aggregation helps the strain to significantly adhere and colonize to the intestinal epithelium, thereby reducing pathogen adhesion [44]. *L. helveticus* LH10 showed auto-aggregation rates of 57.03%, 71.58%, and 79.90% at 3 h, 6 h, and 9 h in saline, respectively, while auto-aggregation rates over 50% are usually considered to meet probiotic features [45]. Therefore, *L. helveticus* LH10 possessed excellent binding properties.

### 3.10. Stress-Resistant Phenotype Analysis

#### 3.10.1. Bile Salt Resistance

The presence of 0.03–0.3% bile salts in the small intestine decreased the growth and metabolism of microorganisms [46], so the bile salt tolerance helped to evaluate whether lactic acid bacteria could survive and further play a probiotic role in the intestinal tract. After incubation in bovine bile salt for 3 h, the viable bacterial count of *L. helveticus* LH10 decreased to 7.42 ± 0.01lg CFU/mL, with a survival rate of 99.73%, indicating that *L. helveticus* LH10 had good tolerance to bile salts and potential applications.

#### 3.10.2. Gastrointestinal Fluid Resistance

**Figure 5:**
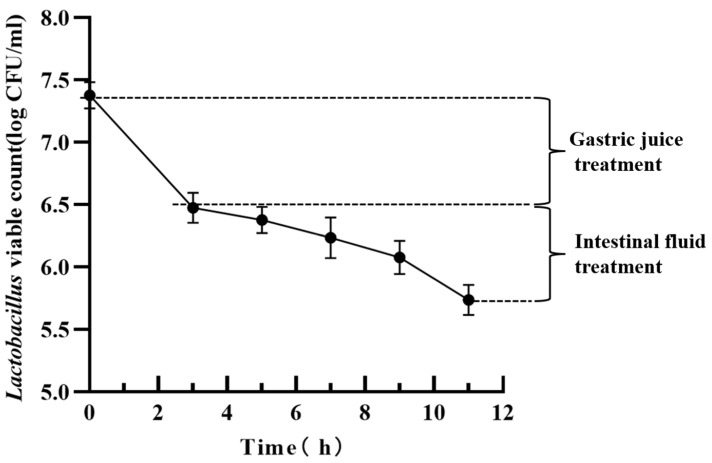
Tolerance of *L. helveticus* LH10 to gastrointestinal fluids. Simulated food digestion process with simulated gastric fluid treatment for the first 3 h and simulated intestinal fluid treatment for the last 8 h.

The tolerance to gastrointestinal fluids is another important index for evaluating probiotic properties. After 3 h of simulated gastric juice incubation, the viable bacterial count of *L. helveticus* LH10 was significantly reduced, and the concentration decreased by 0.74 log CFU/mL. After 8 h of intestinal fluid incubation, the concentration decreased by 0.76 log CFU/mL, which showed a slow decreasing trend. Finally, after 11 h of gastrointestinal fluid incubation, the final concentration of *L. helveticus* LH10 was 5.80 log CFU/mL and the survival rate was 79.45% (Figure 5), indicating that *L. helveticus* LH10 had a good tolerance to the simulated gastrointestinal environment.

### 3.11. Stress-Resistant Genotype Analysis

*Lactobacillus* had many genes related to environmental tolerance, including acid–base, bile salt, temperature, oxygen, and osmolality. According to the literature, the KEGG-annotated genes were searched and summarized (Table 4). *L. helveticus* LH10 possessed a wide range of proteases and chaperone complexes involved in heat stress response, including *clp* proteases, *hslV* proteases, Lon-like proteases, the *groES*, *groEL*, *dnaJ*, *grpe-hrcA* chaperone complex, *HSP20* [47], etc. In addition, it was found that *L.helveticus* LH10 had a potential gene encoding a heat stress protein and two potential genes encoding cold stress proteins, *htpX* and *cspA*.

For acid stress genes, *L. helveticus* LH10 has an intact F_o_F_1_ proton pump ATPase system (atpA-atpH) and a gene for a Na^+^/H^+^ antitransporter protein (a key protein in the acid stress response); *L. helveticus* LH10 also has a gene encoding an alkaline stress protein, *aspS* [47].

Bile salt tolerance in *Lactobacillus* has been reported to be achieved mainly by bile salt hydrolases [48] as well as bile salt transporter proteins [49]. One bile-salt-hydrolase-related gene was detected in *L. helveticus* LH10, and a rich family of transporter proteins was detected. Therefore, the strain could achieve bile salt excretion through bile salt hydrolase and the efflux system of these transporter proteins. The transporter protein families mainly include the MFS family and the ABC transporter protein family; a total of 7 genes related to the MFS transporter proteins and 12 genes related to the ABC transporter protein family were detected in *L. helveticus* LH10, which might reflect that *L. helveticus* LH10 had a good tolerance to bile salts at the genetic level. This was also consistent with the phenotyping experiment.

Finally, an osmolarity-related stress gene, *TC.APA*, and five antioxidant-related genes were also detected in the LH10 genome.

### 3.12. Bacteriostatic Ability

**Figure 6:**
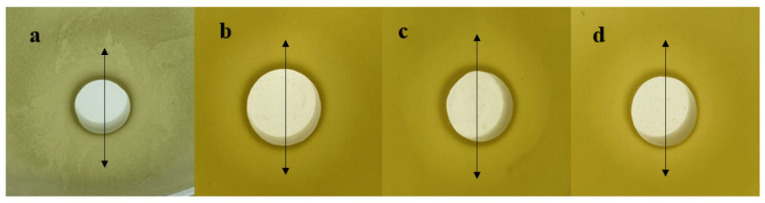
Inhibitory effect of *Lactobacillus helveticus* LH10 against *Staphylococcus aureus* (**a**), *Salmonella enteritidis* (**b**), *Escherichia coli* (**c**), and *Bacillus cereus* (**d**). Each bright circle which was marked with arrows represents the corresponding inhibitory zone.

The inhibition zone diameters of *L. helveticus* LH10 fermented supernatants against *Staphylococcus aureus*, *Salmonella enteritidis*, *Escherichia coli*, and *Bacillus cereus* were 14 ± 0.4 mm, 17 ± 0.5 mm, 18 ± 0.7 mm, and 15 ± 0.4 mm (Figure 6), respectively, indicating *L. helveticus* LH10 had a significant inhibitory effect on all four pathogenic bacteria; especially, *L. helveticus* LH10 had the most obvious inhibitory effect on *Escherichia coli*. The significant inhibitory effect of LH10 could help probiotics to maintain the balance and stability of intestinal flora [50].

### 3.13. Bacteriocin Identification

**Figure 7:**
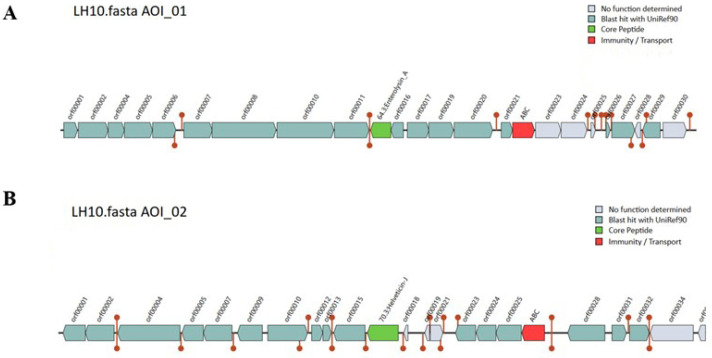
The bacteriocin gene cluster predicted from *L. helveticus* LH10. (**A**) The main Enterolysin_A protein-encoding gene clusters, (**B**) the main helveticin J protein-encoding gene clusters searched for through the BAGEL4 bacteriocin database.

Bacteriocin production increases the competitive advantage of strains in the gut [51]. Two bacteriocin gene clusters related to helveticin J and enterolysin A were respectively identified by BAGEL4, with a match of 60.25% and 44.54% (Figure 7). Helveticin J, encoded by 22 genes, is a large heat-insensitive bacteriocin with a narrow inhibition spectrum, originally isolated from *Lactobacillus helveticus* [52]. Enterolysin_A is a heat-unstable protein produced by *Enterococcus faecalis* LMG 2333, with a broad inhibition spectrum, which disrupts the cell walls of bacteria [53].

## 4. Conclusions

In general, at the gene level, after phenotypic experiments, *L.helveticus* LH10 possessed excellent gastrointestinal environmental tolerance and bacteriostatic ability against four pathogens, and two bacteriocin gene clusters related to Helveticin J and Enterolysin_A in the genome of *L. helveticus* LH10 were found. *L. helveticus* LH10 had some antibiotic resistance, but no plasmids; therefore, its resistance genes could not horizontally transfer. Meanwhile, it did not have virulence factors or hemolytic properties, indicating that it was a potential probiotic candidate. These comprehensive genome analyses and phenotypic experiments provide more clues into the probiotic properties of *L. helveticus* LH10.

## Figures and Tables

**Table 1:** General information of *Lactobacillus helveticus* genomes.

Strains	*Lactobacillus helveticus*			
	LH10	D75	LH5	R0052
Number of scaffolds	1	1	3	1
Genome size (Mb)	1.81	2.05	2.16	2.13
GC (%)	36.6	37.03	36.92	36.80
Coding gene number	2044	2187	2193	2288
rRNA	12	15	12	12
tRNA	63	64	63	61
GenBank	CP149445.1	CP020029.1	CP019581.1	CP003799.1

**Table 2:** Bacteriostatic circle diameter of *L. helveticus* LH10 and antibiotic resistance results.

Category of Drug Sensitivity Test Paper	Name of Drug Sensitivity Test Paper	Judgment Standard of Bacteriostatic Circle Diameter (mm)			Result
		R	I	S	
Lincosamide antibiotics	clindamycin	≤14	15–20	≥21	28 ± 0.7 (S)
β Lactam antibiotics	penicillin	≤19	20–23	≥24	25 ± 0.5 (S)
	ampicillin	≤19	20–23	≥24	22 ± 0.3 (I)
	cefamezin	≤19	20–22	≥23	/(R)
Tetracyclines antibiotics	tetracycline	≤14	15–18	≥19	24 ± 0.3 (S)
Glycopeptides antibiotics	vancomycin	—	—	≥15	/(R)
Sulfonamides antibiotics	sulfamethoxazole	≤10	11–15	≥16	7 ± 0.2 (R)
Aminoglycoside antibiotics	gentamicin	≤12	13–14	≥15	12 ± 0.5 (R)
Macrolide antibiotics	erythromycin	≤13	14–22	≥23	32 ± 0.3 (S)
Quinolone antibiotics	ciprofloxacin	≤15	16–20	≥21	12 ± 0.6 (R)
Rifamycin antibiotics	rifampicin	≤16	17–19	≥20	25 ± 0.4 (S)
Miscellaneous agents	chloramphenicol	≤12	13–17	≥18	22 ± 0.7 (S)

/: no inhibitory zone, S: sensitive, I: intermediate, R: resistant.

**Table 3:** Antibiotic genotype prediction of *L. helveticus* LH10.

Drug Class	Gene Name	Locus_Tag	Definition
Macrolide	*macB*	LH10_GM001871	ATP-binding cassette (ABC) transporter that exports macrolides with 14- or 15- membered lactones
	*carA*	LH10_GM000399	efflux pump complex or subunit conferring antibiotic resistance
	*msr(B)*	LH10_GM000751	efflux pump complex or subunit conferring antibiotic resistance
Glycopeptides	*vanHO*	LH10_GM000065	antibiotic resistance gene cluster, cassette, or operon, determinant of resistance to glycopeptide antibiotics, protein(s) conferring antibiotic resistance via molecular bypass
	*vanSI*	LH10_GM000102	complex or subunit conferring antibiotic resistance, protein(s) and two-component regulatory system modulating antibiotic efflux
	*vanRM*	LH10_GM001853	antibiotic resistance gene cluster, cassette, or operon, determinant of aminoglycoside resistance, determinant of resistance to glycopeptide antibiotics, efflux pump complex or subunit conferring antibiotic resistance
	*vanB*	LH10_GM000161	a D-Ala-D-Ala ligase homolog that synthesizes D-Ala-D-Lac, an alternative substrate for peptidoglycan synthesis that reduces vancomycin binding affinity.
	*vanA*	LH10_GM000161	a D-Ala-D-Ala ligase homolog that synthesizes D-Ala-D-Lac, an alternative substrate for peptidoglycan synthesis that reduces vancomycin binding affinity.
	*vanC*	LH10_GM000161	a D-Ala-D-Ala ligase homolog that synthesizes D-Ala-D-Lac, an alternative substrate for peptidoglycan synthesis that reduces vancomycin binding affinity.
Tetracyclines	*Tet* *(* *Q* *)*	LH10_GM000061	antibiotic target protection protein, determinant of tetracycline resistance
	*tetA* *(* *60* *)*	LH10_GM000817	efflux pump complex or subunit conferring antibiotic resistance
	*tcmA*	LH10_GM000470	efflux pump complex or subunit conferring antibiotic resistance
	*adeR*	LH10_GM001410	positive regulator of AdeABC efflux system
	*tetA (58)*	LH10_GM000159	Tetracycline efflux pump
Peptide	*arnA*	LH10_GM000779	determinant of polymyxin resistance, gene altering cell wall charge
Phosphonic	*mdtG*	LH10_GM001887	efflux pump complex or subunit conferring antibiotic resistance
Diaminopyrimidine	*dfrA26*	LH10_GM001252	antibiotic target replacement protein, determinant of diaminopyrimidine resistance
	*dfrE*	LH10_GM001253	antibiotic target replacement protein, determinant of diaminopyrimidine resistance
Aminoglycoside	*kdpE*	LH10_GM000607	transcriptional activator that is part of the two-component system KdpD/KdpE
Lincosamide	*lmrD*	LH10_GM001591	efflux pump complex or subunit conferring antibiotic resistance
Elfamycin	*Scin_EFTu_ELF*	LH10_GM001297	sequence variants of *Streptomyces* cinnamoneus elongation factor Tu that confer resistance to elfamycin antibiotics

**Table 4:** Stress-related genes of *Lactobacillus helveticus* LH10.

Stress Response	Gene Locus	Gene Name	Definition
Temperature	LH10_GM000282	*clpC*	ATP-dependent Clp protease ATP-binding subunit ClpC
	LH10_GM001466	*clpP*	ATP-dependent Clp protease, protease subunit
	LH10_GM001524	*clpE*	ATP-dependent Clp protease ATP-binding subunit ClpE
	LH10_GM001967	*clpE*	ATP-dependent Clp protease ATP-binding subunit ClpE
	LH10_GM001295	*clpX*	ATP-dependent Clp protease ATP-binding subunit ClpX
	LH10_GM001156	*hslV*, *clpQ*	ATP-dependent HslUV protease, peptidase subunit HslV
	LH10_GM001305	*—*	Lon-like protease
	LH10_GM000217	*HSP20*	HSP20 family protein
	LH10_GM000276	*hslO*	molecular chaperone Hsp33
	LH10_GM000417	*groES*,	HSPE1 chaperonin GroES
	LH10_GM000418	*groEL*,	HSPD1 chaperonin GroEL
	LH10_GM000839	*hrcA*	heat-inducible transcriptional repressor
	LH10_GM000840	*GRPE*	molecular chaperone GrpE
	LH10_GM000841	*dnaK*,	HSPA9 molecular chaperone DnaK
	LH10_GM000842	*dnaJ*	molecular chaperone DnaJ
	LH10_GM001327	*cspA*	cold shock protein (beta-ribbon, CspA family)
	LH10_GM001618	*cspA*	cold shock protein (beta-ribbon, CspA family)
	LH10_GM000122	*htpX*	heat shock protein HtpX
Acid	LH10_GM001377	*ATPF1E*, *atpC*	F-type H+-transporting ATPase subunit epsilon
	LH10_GM001378	*ATPF1B*, *atpD*	F-type H+-transporting ATPase subunit beta
	LH10_GM001379	*ATPF1G*, *atpG*	F-type H+-transporting ATPase subunit gamma
	LH10_GM001380	*ATPF1A*, *atpA*	F-type H+-transporting ATPase subunit alpha
	LH10_GM001381	*ATPF1D*, *atpH*	F-type H+-transporting ATPase subunit delta
	LH10_GM001382	*ATPF0B*, *atpF*	F-type H+-transporting ATPase subunit b
	LH10_GM001383	*ATPF0C*, *atpE*	F-type H+-transporting ATPase subunit c
	LH10_GM001384	*ATPF0A*, *atpB*	F-type H+-transporting ATPase subunit a
	LH10_GM001621	*nhaC*	Na+:H+ antiporter, NhaC family Alkaline LH10_GM001206 aspS aspartyl-tRNA synthetase
Bile salt	LH10_GM000642	*lmrB*	MFS transporter, DHA2 family, lincomycin resistance protein
	LH10_GM001601	*mdtG*	MFS transporter, DHA1 family, multidrug resistance protein
	LH10_GM001887	*mdtG*	MFS transporter, DHA1 family, multidrug resistance protein
	LH10_GM002020	*pbuG*	putative MFS transporter, AGZA family, xanthine/uracil permease
	LH10_GM002024	*pbuG*	putative MFS transporter, AGZA family, xanthine/uracil permease
	LH10_GM002029	*pbuG*	putative MFS transporter, AGZA family, xanthine/uracil permease
	LH10_GM002035	*yaaU*	MFS transporter, putative metabolite transport protein
	LH10_GM000027	*ABC.X2.A*	putative ABC transport system ATP-binding protein
	LH10_GM000028	*ABC.X2.P*	putative ABC transport system permease protein
	LH10_GM000052	*ABC.X4.A*	putative ABC transport system ATP-binding protein
	LH10_GM000053	*ABC.X4.P*	putative ABC transport system permease protein
	LH10_GM000054	*ABC.X4.S*	putative ABC transport system substrate-binding protein
	LH10_GM001761	*ABC.CD.P*	putative ABC transport system permease protein
	LH10_GM001762	*ABC.CD.A*	putative ABC transport system ATP-binding protein
	LH10_GM001871	*ABC.CD.A*	putative ABC transport system ATP-binding protein
	LH10_GM001872	*ABC.CD.P*	putative ABC transport system permease protein
	LH10_GM001970	*ABC.CD.A*	putative ABC transport system ATP-binding protein
	LH10_GM001972	*ABC.CD.P*	putative ABC transport system permease protein
	LH10_GM002007 LH10_GM001256	*ABC.CD.A* *Bsh*	putative ABC transport system ATP-binding protein bile salt hydrolase
Osmotic stress	LH10_GM000804	*TC.APA*	basic amino acid/polyamine antiporter, APA family
Oxidative stress	LH10_GM001866	*nfr2*	flavin reductase (NADH) subunit 2
	LH10_GM000437	*trxA*	thioredoxin 1
	LH10_GM000492	*trxA*	thioredoxin 1
	LH10_GM000494	*trxB*,	TRR thioredoxin reductase (NADPH)
	LH10_GM001520	*spxA*	regulatory protein spx

## Data Availability

Data are contained within the article.
